# G-Quadruplex Structures and CpG Methylation Cause Drop-Out of the Maternal Allele in Polymerase Chain Reaction Amplification of the Imprinted *MEST* Gene Promoter

**DOI:** 10.1371/journal.pone.0113955

**Published:** 2014-12-01

**Authors:** Aaron J. Stevens, Selma Stuffrein-Roberts, Simone L. Cree, Andrew Gibb, Allison L. Miller, Kit Doudney, Alan Aitchison, Michael R. Eccles, Peter R. Joyce, Vyacheslav V. Filichev, Martin A. Kennedy

**Affiliations:** 1 Department of Pathology, University of Otago, Christchurch 8140, New Zealand; 2 Department of Pathology, University of Otago, Dunedin School of Medicine, Dunedin, New Zealand; 3 Department of Psychological Medicine, University of Otago, Christchurch 8140, New Zealand; 4 Institute of Fundamental Sciences, Massey University, Palmerston North, New Zealand; Florida State University, United States of America

## Abstract

We observed apparent non-Mendelian behaviour of alleles when genotyping a region in a CpG island at the 5′ end of the maternally imprinted human *MEST* isoform. This region contains three single nucleotide polymorphisms (SNPs) in total linkage disequilibrium, such that only two haplotypes occur in the human population. Only one haplotype was detectable in each subject, never both, despite the use of multiple primers and several genotyping methods. We observed that this region contains motifs capable of forming several G-quadruplex structures. Circular dichroism spectroscopy and native polyacrylamide gel electrophoresis confirmed that at least three G-quadruplexes form *in vitro* in the presence of potassium ions, and one of these structures has a *T*_m_ of greater than 99°C in polymerase chain reaction (PCR) buffer. We demonstrate that it is the methylated maternal allele that is always lost during PCR amplification, and that formation of G-quadruplexes and presence of methylated cytosines both contributed to this phenomenon. This observed parent-of-origin specific allelic drop-out has important implications for analysis of imprinted genes in research and diagnostic settings.

## Introduction

DNA can adopt several structural alternatives to the canonical double helix (B-DNA) and such structures are emerging as important signals for many processes relevant to genome function and cellular biology. One of the most prevalent and intriguing non B-DNA structures is the G-quadruplex (G4), which was first described in immunoglobulin switch regions and telomeric DNA at the ends of chromosomes [1], [2]. Guanine is unique among the nucleoside bases found in DNA due to its potential to form stable, self-associating structures through hydrogen bonding [3]. These structures, each containing four guanine bases and known as a G-quartet, can form within or between DNA strands under certain ionic conditions, and a stack of G-quartet forms the G4 [1], [2], [4]. Because G4 formation is dependent on guanine base repetitions, regions of linear DNA sequence likely to form G4 can be predicted by relatively straightforward algorithms [5]–[9].

G4 structures have many *in vivo* functions [10]–[12], but are also of interest because of their effect on *in vitro* processes. The potential for G-quadruplex structures to inhibit DNA synthesis has long been recognized [13], [14]. In the clinical setting, diagnostic failure by allelic drop-out during PCR can lead to false positive or negative diagnoses and at least some instances of this can be attributed to differential G4 formation. For example, allelic drop-out was documented for two cases of Rett syndrome in which rare, benign variants appeared to be homozygous, and preferential amplification of one allele occurred due to disruption of G4 structures by the variants [15]. A similar mechanism of allelic drop-out was also observed in molecular diagnosis of multiple endocrine neoplasia I [16]. In these reports the variants were found to impact on G4-motifs or the related i-motif, which can form on the corresponding C-rich strand, and the allelic drop-out was dependent on the presence of a cation (potassium or magnesium) known to be essential for G4 structure. A significant proportion of variants involved in human disease may be prone to this problem [16].

G4 structures are found in the promoters of many genes and these regions are often enriched for the dinucleotide 5′-CpG-3′, which is the substrate for DNA methyltransferases. Methylation of cytosines at such sites is an important and dynamic regulator of transcription, and indeed inappropriate methylation has proven to be an important factor in development and progression of tumours [17]. DNA methylation also plays an important role in gene regulation and gene silencing, modifying gene expression in response to many internal and external environmental stimuli [18], [19]. Little is known about the relationship between cytosine methylation in DNA and the formation or function of G4 in these regions.

Early work described stabilisation of G4 through cytosine-cytosine base pairing, an effect that was greatly enhanced by cytosine methylation [20], and showed that DNA methyltransferase has an exceptional ability to methylate unusual DNA structures [21]. More recently, it was observed that CpGs within G4-forming regions are rarely methylated, suggesting a functional relationship that generally precludes co-location of methylated CpG and G4 motifs [22]. Despite this under-representation of methylated CpGs in G4-forming regions, G4 motifs do still occur in the genome at sites that are methylated. For example, the CpG island of the oncogene *BCL2* contains G-rich regions capable of forming a G4 structure, and methylation of cytosines within the G4 motif markedly stabilises the G4 [23].

Genomic imprinting is an epigenetic form of gene regulation involving differential cytosine methylation of alleles, which results in mono-allelic transcription of certain autosomal genes determined by their parental origin [24]. Imprinted loci play important roles in development, and loss of imprinting at these loci can lead to various diseases [25]. This report concerns a region upstream of the imprinted promoter for the human mesoderm-specific transcript (*MEST*) gene, previously known as paternally expressed gene 1 (*PEG1*), a gene which is involved in development and maternal behaviour [26]–[28]. *MEST* has two main isoforms, one of which displays genomic imprinting and mono-allelic expression, and the other of which, transcribed from a distal upstream promoter, displays bi-allelic expression [29]. The focus of this report is a region within the CpG island of the imprinted isoform, the maternal, non-expressed allele of which displays heavy methylation at CpG dinucleotides [30]–[32]. We describe unusual *in vitro* properties of this region including the formation of at least three G4 structures, a consistent and robust parent-of-origin specific allelic drop-out during PCR, and the contribution of both G4s and DNA methylation to this phenomenon.

## Materials and Methods

### Genomic DNA samples

SNP discovery and analysis of inheritance patterns was carried out in subjects drawn from a family study of major depression and its treatment, called the Genetics and Pharmacogenetics of Depression Study (GODS) [33]. Subjects in this cohort were recruited on the basis that they had been treated for major depression and had at least two first-degree relatives (parents and/or siblings) who were willing to participate. The collection of DNA for GODS was approved by the Canterbury Ethics Committee (Christchurch, New Zealand; approval number CTY/00/12/176). All participants were provided with an information sheet and verbal explanation of the study, and written consent was then obtained. Participants were all adults.

### Polymerase chain reaction (PCR)

Unless otherwise indicated, PCR was performed with Fisher Taq-ti polymerase (Fisher Biotec, Wembley WA, Australia) using an initial denaturation step of 95°C for 2 min, followed by 35 cycles of 15 sec denaturation (95°C), 30 sec annealing (63°C) and 45 sec extension (72°C) and a final extension of 72°C for 4 min. Oligonucleotides (Table S1) were sourced from Integrated DNA Technologies (IDT Pte. Ltd., Singapore), and deoxynucleoside triphosphates from Fisher Biotec (Wembley WA, Australia). Where specified, 7-deaza-2′-deoxyguanosine 5′-triphosphate (7-deaza-dGTP; Trilink Biotechnologies, San Diego, USA) was included in a 1∶3 ratio with dGTP. Betaine was included in all PCRs at 1 M final concentration, because it was found to significantly improve PCR efficiency (greater yield and much cleaner amplification of products).

### DHPLC analysis and genotyping

Mutation analysis and initial genotyping of the *MEST* region was carried out on a WAVE Fragment Analysis System (Transgenomic, Omaha, NE, USA), which used denaturing high performance liquid chromatography (dHPLC) and detection by UV absorption to distinguish the melting profiles of homoduplex or heteroduplex fragments generated by PCR [34]. Following PCR amplification of the region of interest, the products were repeatedly heated (from a starting temperature of 95°C) and cooled over 45 cycles, with each cycle 1.5°C cooler than the previous one. This allowed the formation of hetero- and homoduplexes in those samples that are heterozygous at a specific site. Addition of known homoduplex PCR products to unknown homoduplexes prior to WAVE analysis was used to characterize homozygote (or hemizygote) amplicons for different alleles.

### Sanger DNA sequencing

Sanger DNA sequencing was carried out on PCR products purified using AcroPrep (PALL Corporation, New York, USA) 96 well filter plates (omega 30K) then resuspended in water. Purified PCR amplicon (∼10 ng) was sequenced with the appropriate primer using BigDye Terminator v3.1 Cycle Sequencing Kit (Applied Biosystems, Foster City, CA, USA), following the manufacturer's protocol. Sequencing reaction products were run on an AB3130*xl* fragment analysis system equipped with a 50 cm capillary using POP7 polymer.

### Non-denaturing polyacrylamide gel electrophoresis (PAGE)

Non-denaturing PAGE was carried out on a 15% polyacrylamide gel (40% bis-acrylamide/bis solution 37.5∶1 Bio Rad Laboratories, Inc) in TBE (89 mM Tris-borate pH 8.3, 2 mM EDTA) containing 100 mM KCl. Sample loading buffer (4% sucrose in TBE plus 100 mM KCl) containing 10 µM of oligonucleotide was heated at 95°C for 5 minutes then cooled slowly to room temperature (22°C) overnight. Gels were run for 40 minutes at 200 mAh/200 V/37 W, maintained below 25°C. Molecular weight markers consisted of custom synthesised oligothymidylates of 10, 20, 30, 40 and 50 nucleotides in length (IDT Pte. Ltd., Singapore). After electrophoresis, gels were visualised by UV shadowing against a thin layer chromatography plate followed by staining with SYBR Safe and destaining in water.

### Circular Dichroism Spectroscopy (CD)

Oligomers were purchased from Integrated DNA Technologies (IDT Pte. Ltd., Singapore). Unless stated otherwise all experiments were carried out in 10 mM NaPi buffer with 50 mM KCl. Where possible all ionic concentrations were comparable to concentrations present in the Tris-based PCR buffer (Roche, Mannheim, Germany) in which the initial genotyping failure was observed, i.e. 1.5 mM Mg^2+^ and 50 mM KCl. 4 µM of oligonucleotide was heated at 95°C for 10 minutes then cooled slowly to room temperature (22°C) overnight, for CD analyses the following day.

CD measurements and CD melting studies were performed on a J-815 CD Spectrometer (Jasco Analytical Instruments, MD, USA), with a 1 mm path length quartz cuvette. Sample temperature was regulated with a Peltier controller. CD spectra were collected across 340 nm to 200 nm in 1 nm increments and the reported spectra corresponded to the average of at least three scans. The conformation of the motif was determined by spectral investigation where the oligonucleotide is 100% helical. CD melting curves were obtained by monitoring the change in ellipticity as a function of temperature across all wavelengths. Temperature was increased from 25°C to 95°C at a rate of 0.25°C min^−1^. Analyses of the CD melting curves yielded *T*_m_ at 260 nm, calculated as the temperature at which the structure was 50% folded in solution. An appropriate buffer blank correction was made for all spectra.

### Bisulfite Conversion

For investigation of DNA methylation, genomic DNA was subjected to bisulfite conversion using an EZ DNA Methylation-Gold Kit (Zymo Research, Irvine, CA), according to the manufacturer's recommendations. This involved treatment of genomic DNA with CT conversion reagent for 2.5 hours at 64°C, followed by size exclusion centrifugation on an AcroPrep 96 well filter plate (PALL Corporation, New York, USA; omega 30K). The final product was resuspended in TE and quantified on a NanoDrop2000 (Thermo Scientific, Wilmington DE, USA).

### Methylation-specific PCR

This method was similar to that described by Herman *et al.* [35]. Primers were designed with 5′ homology to either converted (TG) or protected (CG) dinucleotides of bisulfite-treated DNA (BSPF1CG and BSPF1TG, Table S1). In conjunction with a common reverse primer (BSPMESTR3, Table S1), this allowed selective amplification of paternal (bisulfite converted) and maternal (methylation protected) alleles in separate PCR procedures. The PCR was performed using EpiTaq HS (TaKaRa, Shiga, Japan) in a total volume of 50 µl containing Takara Mg^2+^ free PCR buffer, 2.5 mM MgCl_2_ (Takara), 0.4 µM of each primer, 200 µM dNTPs (2.4 mM each), 1 M betaine, 1 U Takara Epitaq HS enzyme (Takara) and ∼30 ng of genomic DNA. Standard thermal cycling conditions consisted of an initial denaturation step of 94°C for 20 sec, 15 cycles starting at 60°C with a temperature decrease of 1°C per cycle; followed by 35 cycles of 94°C for 15 sec, 45°C for 15 sec and 68°C for 45 sec with a final extension of 68°C for 5 min.

### In vitro DNA methylation

*In vitro* methylation and digestion experiments were performed on PCR product generated from genomic DNA or from the synthetic gBlocks templates (IDT Pte. Ltd., Singapore) described below, using enzymes purchased from New England Biolabs Inc, (Ipswich, MA, USA). *In vitro* methylation was carried out by incubation with M.SssI for 120 minutes at 37°C followed by heat inactivation at 65°C for 20 minutes, as recommended by the manufacturer's protocol. Each DNA sample was then incubated with the restriction enzyme MspI and its methylation-sensitive isoschizomer HpaII, to assess the extent of *in vitro* methylation. The resulting digestion products were analysed on a MultiNA (Shimadzu, Kyoto, Japan) microchip electrophoresis system.

Where methylation-dependent digestion was required, this was performed on ∼70 ng genomic DNA using McrBC endonuclease (New England Biolabs Inc., Ipswich, MA, USA) according to the manufacturer's protocol. PCR was then performed on McrBC digested genomic DNA using primers MESTPF1/MESTPR4 (Table S1) and the amplicon was analysed by Sanger sequencing. The genotyping results obtained in this way were compared to those generated from PCR products derived from untreated genomic DNA of known haplotypes.

*In vitro* methylation was also carried out as described above on templates containing 7-deaza-dGTP. To ensure that adequate methylation of these templates had occurred, they were subjected to bisulfite conversion and sequencing as described above.

### Modelling allele specific drop-outs

To examine the effect of methylation on allelic drop-out we simulated the situation for genomic DNA by using *in vitro* methylation of templates representing the two *MEST* haplotypes. PCR products were generated from genomic DNA of two GODS subjects known to give different haplotypes, using KAPA 2GRobust enzyme (Kapa Biosystems, Inc., Woburn, MA, USA) and primer combination MESTPF1/MESTPR6 (Table S1) following the manufacturer's protocol for G-rich DNA. Products were then subjected to *in vitro* methylation with M.SssI CpG methyltransferase, and the effectiveness of methylation was tested by enzymatic digestion (as described above). These products were purified and diluted to 2 ng/ul in TE, then different combinations were mixed at equal concentrations to provide synthetic templates modelling different allelic methylation states of genomic DNA. PCR amplification was performed on mixed templates using primers MESTPF1/MESTPR3 (Table S1) and the resulting amplicons were purified and sequenced as above.

To experimentally differentiate between the contribution of methylation and G4 formation in allelic drop-out, two customized gBlocks were synthesized (IDT Pte. Ltd., Singapore). The wild-type gBlock contained a fragment spanning 636 bp of the human imprinted *MEST* promoter, and encompassed all three SNPs (of the ATA haplotype) and all three quadruplex forming regions (Figure S1). The mutant gBlock represented the equivalent region, except that T bases were substituted for all G bases expected to be involved in G-quartet formation for the three predicted G4s (Figure S1). *In vitro* methylation was performed as described above on PCR products amplified from both synthetic gBlock templates with primers MESTPF1/MESTPR3C (Table S1). These products were purified and diluted to 2 ng/ul in TE, then semi-nested PCR was performed on various mixed templates of equal concentration, using primers MESTPF1/MESTPR3 (Table S1). The resulting amplicons were purified and sequenced as above.

### Bioinformatic analyses

Predictions of putative G4 motifs were made using the software algorithms Quadparser [5] G4P_calculator.exe [6], QGRS Mapper [7], Quadfinder [8], QGRS Predictor [9]. Where necessary, both strands were analysed separately to ensure G4-motifs were detected in both orientations. Primer design, analysis of sequence alignments, and DNA sequencing results were managed using Geneious 5.5.3 software (Biomatters Ltd, Auckland, New Zealand).

## Results

### Apparent non-Mendelian behaviour of MEST promoter SNPs

**Figure 1:**
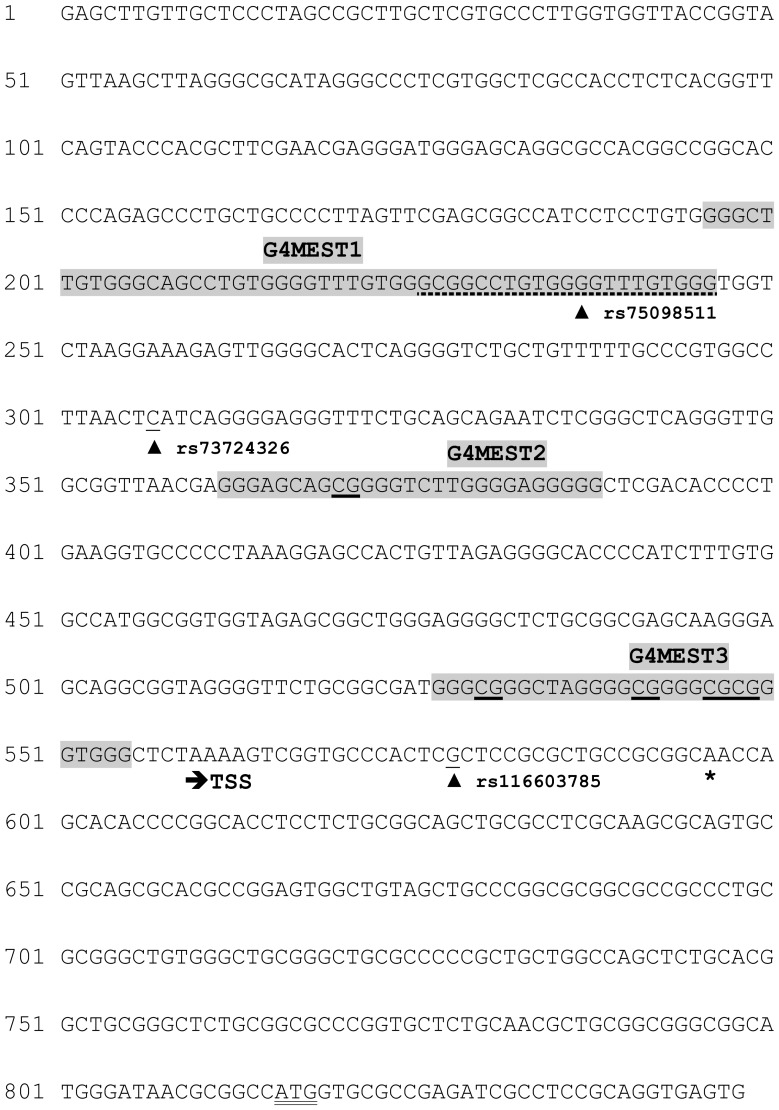
Sequence of MEST promoter region indicating key features. This figure encompasses the hg19 coordinates chr7:130131340-130132187. Features indicated are the three G4s (grey shading, with the extended G4MEST1L region indicated by a dashed underline), the three SNPs (arrowheads with rs IDs as indicated), CpG dinucleotides within the G4 regions (underlined), and the transcription start site (TSS, arrowed) and start codon (double underline) for the imprinted isoform of *MEST* (NM_001253900). The most 5′ base of the 381bp region studied by Bunyan *et al*. (2011)[37] is indicated by an asterisk.

While exploring genetic variability in the human imprinted gene *MEST* we encountered three SNPs in a CpG island located immediately 5′ of the imprinted MEST isoform [36] (Figure 1). These SNPs (rs75098511, rs73724326, rs116603785) displayed total linkage disequilibrium such that only two haplotypes (named ATA or GCG) encompassing the three SNPs exist [36]. When these SNPs were genotyped in many subjects we observed a non-Mendelian pattern whereby only one apparently homozygous haplotype was detectable in each subject, and no heterozygotes were observed (Table S2). This absence of heterozygotes occurred despite the use of several different enzyme and buffer combinations for PCR (including addition of betaine or DMSO at various concentrations), and several different methods for genotyping including DHPLC (Transgenomic WAVE Fragment Analysis System), Sanger DNA sequencing, and high resolution melt (HRM) analysis on an Eco real time PCR instrument (Illumina, San Diego, CA). Three different pairs of primers (MESTPF1/MESTPR2, MESTPF3/MESTPR4, MESTPF5/MESTPR6; Table S1), which generated overlapping amplicons encompassing one or more of the three SNPs, gave similar genotype patterns. This excluded trivial causes of allelic drop-out such as polymorphisms underlying a primer. The frequency at which the respective genotype patterns occurred in the subset of mainly Caucasian subjects examined was 87% for GCG and 13% for ATA [36](Table S2).

Experiments in which genomic DNA from different subjects was mixed prior to PCR proved that these assays were capable of detecting both haplotypes simultaneously [36] indicating that the anomalous genotypes were attributable to some property of the input genomic DNA (Figure S2). We were able to show heterozygosity for SNPs respectively ∼1.7 kb upstream and ∼1 kb downstream of the region of interest, and in the 3′ untranslated region of MEST (Table S2), indicating that this phenomenon was restricted to a relatively narrow region of the *MEST* locus.

The absence of observed heterozygotes for the three promoter SNPs was most likely due to drop-out of one allele during PCR. We excluded the presence of polymorphisms within primer binding sites, a common cause of allelic drop-out, but allelic drop-out can also be caused by the presence of unusual structural features of DNA, including the formation of secondary DNA structures such as G4s [13], [15], [16]. Furthermore, differential methylation, as encountered at imprinted loci like *MEST*, is also reported to cause differential allelic amplification [37], [38]. Therefore we explored the possible contributions of both G4 formation and CpG methylation to the observed allelic drop-out in the *MEST* promoter region.

### G-quadruplex structures in the MEST promoter region

First we sought evidence for regions within the PCR amplicon (Figure 1) capable of forming G4 structures. We used several algorithms for detecting G4 motifs [5]–[9]. There was good agreement across all programmes, although the output from QGRS mapper [7] was selected as the preferred configuration. Three clear G4 motifs, all on the same strand, were detected (Figure 1), with only weak G4 predictions for the complementary strand. We named these regions G4MEST1-3. Subsequent to this initial analysis we recognised that G4MEST1 was located in a longer G-rich region which included the SNP rs75098511 and one CpG dinucleotide (Figure 1). This longer region (called G4MEST1L) was used below for CD analysis of the effects of methylation on *MEST* G4 structures.

### Physical evidence of G4

**Figure 2:**
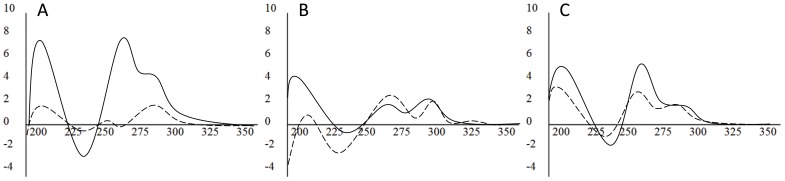
Circular dichroism spectroscopy of putative MEST promoter region G4s. CD spectra of G4MEST1 (A), G4MEST2 (B), and G4MEST3 (C). Molar ellipticity (x10^5^ deg.cm^2^.dmol^−1^) is on the vertical axis and wavelength (nm) is on the horizontal axis. Solid lines represent the CD spectra at 25°C in the presence of 50 mM KCl and dashed lines represent CD spectra in the presence of 50 mM NaCl.

Oligonucleotides spanning G4MEST1, 2 and 3 were synthesised and tested using circular dichroism (CD) spectroscopy for their ability to form G4 *in vitro*. Each region displayed an elliptical minimum of 245 nm and a maximum at 260 nm with a broad shoulder to 295 nm (Figure 2). This spectral profile suggests mixed species in solution, with the dominant conformation for each most likely consisting of parallel structures. Elliptical maxima were highest in the presence of 50 mM KCl, suggesting formation of structures was dependent on the presence of potassium ions, as would be expected for G4 formation.

**Figure 3:**
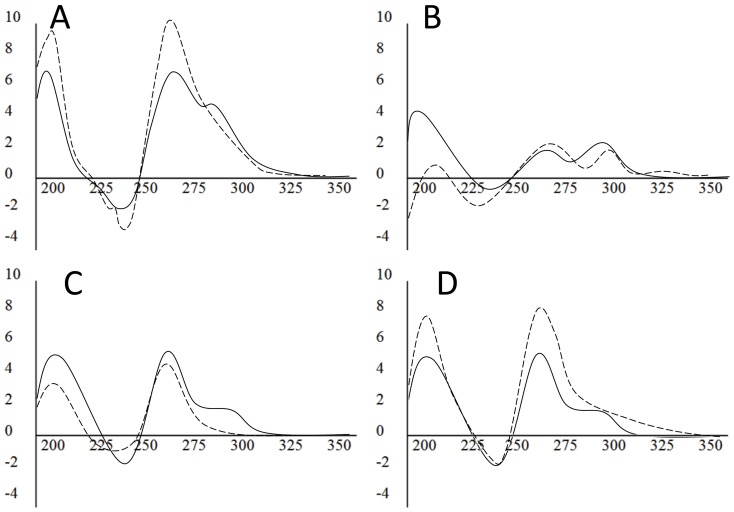
Comparison of G4 conformation in different buffers. CD spectra for G4MEST1L (A), G4MEST2 (B), and G4MEST3 (C) in NaPi, 50 mM KCl (solid line) or PCR buffer (dashed line). (D) CD spectra for G4MEST3 in NaPi, 50 mM KCl (solid line) or NaPi containing 50 mM KCl and 1.5 mM Mg^2+^ (dashed line). Molar ellipticity (x10^5^ deg.cm^2^.dmol^−1^) is on the vertical axis and wavelength (nm) is on the horizontal axis.

We compared structural conformations in PCR buffer and NaPi (containing 50 mM KCl) and observed strong spectral shifts in PCR buffer to a peak at 260 nm and a trough at 245 nm, suggesting complete parallel conformation for G4MEST1 and G4MEST3, but not G4MEST2 (Figure 3). This spectral shift could be replicated for G4MEST1 and G4MEST3 simply by adding 1.5 mM Mg^2+^ to the NaPi buffer (Figure 3D). Therefore we attribute the observed effects of PCR buffer on G4 conformation to the presence of magnesium ions. Further analyses of the observed *T_m_* of G4MEST1-3 indicated that although Mg^2+^ favours parallel conformation, the combination of Tris, KCl and MgCl_2_, as present in PCR buffer, appears to have a significant stabilising impact on G4 structures (Table 1).

**Table 1:** G4 melting temperatures in different buffers, *T_m_* (°C).

Oligonucleotide	PCR buffer	NaPi Buffer	NaPi + MgCl_2_	Sequence^c^
	(50 mM KCL, 1.5 mM MgCl_2_)	(50 mM KCl)	(50 mM KCL, 1.5 mM MgCl_2_)	
**G4MEST1**	56.5	54.0	NA^a^	GGGCTTGTGGGCAGCCTGTGGGGTTTGTGG
**G4MEST1L**	74.0	60.0	76	GGGCTTGTGGGCAGCCTGTGGGGTTTGTGGGCGGCCTGTGGAGTTTGTGGG
**G4MEST1LM**	74.0	57.0	74	GGGCTTGTGGGCAGCCTGTGGGGTTTGTGGGCGGCCTGTGGAGTTTGTGGG
**G4MEST2**	>99.0	57.5	>99	GGGAGCAGCGGGGTCTTGGGGAGGGGG
	85.0^b^		82^b^	
**G4MEST2M**	95.0	Non-G4 spectrum	99	GGGAGCAGCGGGGTCTTGGGGAGGGGG
	79.0^b^		85^b^	
**G4MEST3**	>99.0	80.0	80.0	GGGCGGGCTAGGGGCGGGGCGCGGGTGGG
**G4MEST3M**	92.0	Non-G4 spectrum	70.0	GGGCGGGCTAGGGGCGGGGCGCGGGTGGG

a NA – not available, lacks CpG positions.

b Measurements at 290 nm (all other measurements at 260 nm).

c Methylated cytosines are underlined.

The specificity of CD spectra was demonstrated by performing CD on equivalent control oligonucleotides in which specific G nucleosides contributing to the predicted G4s were replaced by substitution with A nucleosides. None of these oligonucleotides (called G4MEST1A-3A, Table S1) gave CD spectra with G4 characteristics.

### Effect of cytosine methylation on G4 formation

The MEST gene is maternally imprinted, meaning that the allele derived from the mother is heavily methylated at CpG dinucleotides. The 550 bp region (MESTPF1/MESTPR3C amplicon) encompassing the three G4 motifs is 40% GC, and contains 37 CpG dinucleotides. The oligonucleotides G4MEST1L (but not G4MEST1) and G4MEST2 each contain one CpG, and G4MEST3 contains four CpGs (Figure 1), which could potentially be methylated. Oligonucleotides corresponding to these regions were synthesized such that each contained a single 5-methylcytosine residue. These oligonucleotides were named G4MEST1LM, G4MEST2M and G4MEST3M (Table S1). For G4MEST3M we chose to include 5-methylcytosine at only one of the four available CpGs, and this site was chosen because it is most likely to be involved in formation of a G-quartet. The physical properties of these methylated oligonucleotides were then examined to explore the potential impacts of cytosine methylation within G4 motifs.

**Figure 4:**
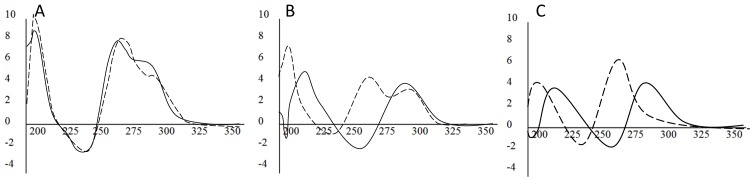
Comparison of methylated G4 conformation in different buffers. CD spectra for methylated oligonucleotides G4MEST1LM (A), G4MEST2M (B), and G4MEST3M (C) in NaPi, 50 mM KCl (solid line) or PCR buffer (dashed line). Molar ellipticity (x10^5^ deg.cm^2^.dmol^−1^) is on the vertical axis and wavelength (nm) is on the horizontal axis.

G4MEST1LM was the only oligonucleotide of the three that gave a G4-specific CD signature in both NaPi and PCR buffers (Figure 4A). In contrast, G4MEST2M in NaPi gave a trough at 265 nm and a peak at 280 nm despite the presence of 50 mM KCl in solution, which is not characteristic of G4 structures; and in PCR buffer it showed a trough at 230 nm and two peaks, one at 260nm and one at ∼290 nm (Figure 4B). This is suggestive of mixed parallel and antiparallel G4 species. G4MEST3M in NaPi gave a very similar spectrum to G4MEST2M, and in PCR buffer it showed a trough at 245 nm and a peak at 260 nm, which is typical of parallel G4 conformation (Figure 4C).

**Figure 5:**
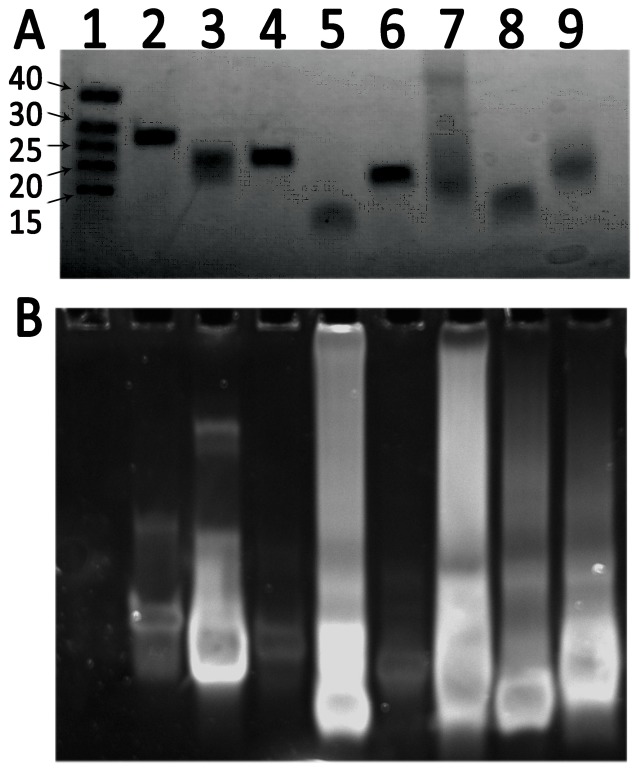
PAGE analysis of G4 oligonucleotides. (A). Non-denaturing 15% PAGE of G4 forming oligonucleotides run in the presence of 100 mM KCl, and visualised using UV shadowing. Lane 1: Oligo-dT size markers (size in bases indicated to left). Lane 2: G4MEST1A (mutant). Lane 3: G4MEST1 (wild-type). Lane 4: G4MEST2A (mutant). Lane 5: G4MEST2 (wild-type). Lane 6: G4MEST3A (mutant). Lane 7: G4MEST3 (wild-type). Lane 8: G4MEST2M (methylated). Lane 9: G4MEST3M (methylated). (B). Same gel stained with SYBR Safe, a dsDNA specific, intercalating dye. Lane numbering as above. Note the oligo-dT size markers are single stranded and therefore not visible.

Non-denaturing PAGE was used to validate the structural properties of the three G4MEST regions (Figure 5). G4MEST1A-3A were used as control oligonucleotides that were not expected to form G4 structures. PAGE showed that the wild-type sequences (G4MEST1-3) migrated with significantly greater mobility than the mutated forms (G4MEST1A-3A), as would be expected due to the increased electrophoretic mobility of the more compact structures arising from G4 formation [1]. G4MEST2 (27 nt), despite showing a relatively weak CD signature (Figures 2-4), displayed the highest mobility, which may indicate the ability to form a very compact secondary structure. The mobility of two methylated oligonucleotides (G4MEST2M and G4MEST3M) was also examined, and these showed similar mobility to their wild-type, unmethylated counterparts. The methylated oligonucleotides G4MEST2M and G4MEST3M had an apparent electrophoretic mobility of 15 and 20 nucleotides (respectively) for sequences of 27 and 29 nucleotides in length (Figure 5A).

Staining of these electrophoretic gels with the intercalating DNA dye SYBR Safe (Life Technologies, Carlsbad,CA, USA) caused fluorescence predominantly of those oligonucleotides expected to form G4 structures (Figure 5B). The staining also suggested the presence of higher order, slower migrating material in lanes containing the wild-type and methylated G4 forming oligonucleotides, but not the mutant forms or oligo-dT size marker.

### Allelic drop-out in PCR with templates methylated in vitro

**Figure 6:**
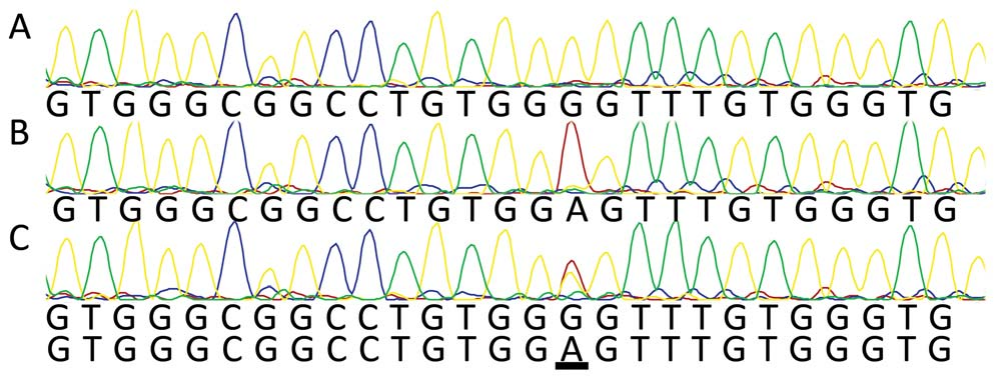
Allelic drop-out in PCR with templates methylated *in vitro*. PCR products (primers MESTPF1/MESTPR6) were generated from genomic DNA homozygous for either the ATA or GCG haplotypes. Methylated and unmethylated forms of these amplicons were diluted and mixed, subjected to PCR, and then genotyped by Sanger sequencing. All three SNPs showed the same pattern, but results for only SNP rs75098511 are illustrated: (A) methylated ATA vs. unmethylated GCG showing apparent GCG homozygosity; (B) methylated GCG vs. unmethylated ATA showing apparent ATA homozygosity; (C) unmethylated ATA vs. unmethylated GCG showing apparent heterozygosity.

To examine the impact of methylation on allelic drop-out in PCR of the *MEST* promoter region we used primers MESTPF1/MESTPR6 to generate 872bp PCR amplicons spanning all three SNPs in this region (Figure 1). Genomic DNA samples known to yield different haplotypes (either ATA or GCG) were used as templates, and PCR products were verified with Sanger sequencing before their use in the next step. PCR amplicons lack DNA methylation or other modifications, and so these ATA or GCG amplicons were identical other than the allelic differences at the three SNPs. *In vitro* methylation of each amplicon was carried out with CpG methyltransferase M.SssI, to produce four different templates: ATA methylated, ATA unmethylated, GCG methylated, and GCG unmethylated. These synthetic templates were mixed in various combinations and used to seed PCR reactions, the products of which were then genotyped by Sanger sequencing. These experiments demonstrated that the methylated allele always dropped out of the PCR regardless of which haplotype was methylated (Figure 6). Furthermore, mixing of both unmethylated templates (ATA or GCG), resulted in clear heterozygous genotypes which confirmed that cytosine methylation of the template is a critical component of the drop-out (Figure 6C).

These *in vitro* mixing experiments were repeated with pairs of templates in which 7 deaza dGTP (Trilink Biotechnologies, San Diego, USA) was incorporated, which prevents formation of Hoogsteen bonds between guanine bases. For all such template pairs, incorporation of 7 deaza dGTP alleviated allelic drop-out of the methylated allele. However, the use of 7 deaza dGTP as a substitute for dGTP during PCR did not alleviate the drop-out thus demonstrating that Hoogsteen bonding in the template DNA was necessary for this phenomenon.

### Effective genotyping of the MEST promoter region

To clarify the factors leading to consistent allelic drop-out at this locus, we attempted to develop a PCR approach that would yield consistent and accurate genotypes from genomic DNA samples. We initially tried to amplify this region with a variety of different thermostable DNA polymerases, as well as one isothermal polymerase, TwistAmp Basic (TwistDX Ltd, Cambridge, UK), but these attempts met with either failure to amplify at all, or allelic drop-out remained a problem.

We reasoned that the cluster of G4 structures in this region would make it difficult to amplify the full region encompassing all three SNPs, and of the three G4s, CD analysis showed G4MEST1 had a predicted *T_m_* of below 60°C in NaPi buffer (Table 1). Therefore, we focused the PCR on a 218bp region that included G4MEST1 and two of the three SNPs. The PCR primers MESTPF1 and MESTPR4 (Table S1) were designed to have an optimal annealing temperature of 63°C, above the predicted *T_m_* of G4MEST1. This amplicon had an overall GC content of 36%, including 15 CpGs. We devised a PCR buffer lacking potassium, which consisted of 2.5 mM sodium phosphate and 2.0 mM MgCl_2_ pH 8.0. Although this buffer proved adequate for generating heterozygotes with the 218 bp amplicon, it was not effective for amplicons of larger than ∼300 bp.

### The maternally imprinted methylated allele drops-out of PCR

Using the above procedure for PCR of a 218 bp amplicon in potassium-free NaPi buffer, we performed genotyping on parent-offspring trios from the GODS cohort. By screening ∼80 offspring we identified three informative trios that consisted of heterozygous offspring, GCG homozygous mothers and heterozygous fathers. Genotyping of these trios demonstrated that in all cases it was the maternal allele which was susceptible to drop-out in normal PCR conditions (Figure S3).

We sought to validate this result by performing methylation-specific PCR on bisulfite treated DNA. One allele of imprinted loci is often heavily methylated, and this is known to be the case for the maternal allele of MEST [28], [31], [32]. Allele-specific amplification based on the methylation status of the underlying DNA can be achieved by bisulfite treatment to convert all unmethylated Cs to Ts [35]. PCR primers were designed with 3′ homology to either converted (TG) or protected (CG) dinucleotides of bisulfite treated DNA. This allowed selective amplification of paternal (bisulfite converted) and maternal (methylation protected) alleles in separate PCR procedures. Genotyping of 20 subjects using this method, including members of the trios examined previously, confirmed that it is indeed the maternal allele which is consistently susceptible to drop-out during PCR.

As a final method for investigating the potential influence of methylation during the observed allelic failure we treated genomic DNA prior to PCR with either the methylation-dependent endonuclease McrBC, which cuts at every methylated CpG dinucleotide, or the methylation-sensitive endonuclease HpaII. After digestion the DNA was amplified by PCR and Sanger sequenced. This enabled the amplification and genotyping of each parental allele through separate reactions, and again confirmed that the maternal methylated allele was lost during genotyping in standard PCR.

### Relative impact of quadruplex versus methylation on allelic drop-out

We have shown that the *MEST* promoter region is capable of forming three stable G4 structures *in vitro*, and that allelic drop-out in PCR involves the maternal, methylated allele. However, it was not clear whether either formation of G4 or DNA methylation alone was sufficient for allelic drop-out, or whether both factors were required. Therefore, we attempted to evaluate the respective impact of each factor on allelic drop-out. Custom gBlocks (IDT, Singapore) of 636 bp in length were synthesised that contained either the wild-type ATA haplotype or an equivalent sequence but with 38 G to T substitutions to remove all potential G4 forming ability (Figure S1). These plasmids were used as PCR templates to generate amplicons for the following series of experiments (Figure S4). As with previous *in vitro* methylation experiments, we used M.SssI to methylate these amplicons. To ensure no residual unmethylated plasmid template contaminated the amplicons, they were treated prior to use with the methylation sensitive enzyme HpaII.

When the methylated wild-type product was mixed with unmethylated mutant product the wild-type allele consistently failed to amplify. Similarly, when the unmethylated wild-type and methylated mutant templates were mixed, only the methylated mutant allele consistently amplified, although occasional weak apparent heterozygosity was observed for this pair of templates. When both methylated templates were mixed, the wild-type template exhibited consistent drop-out. When unmethylated wild-type and unmethylated mutant templates were mixed, PCR usually resulted in a heterozygous genotype, but the wild-type (G4 forming) template was often prone to failure (Figure S4).

**Figure 7:**
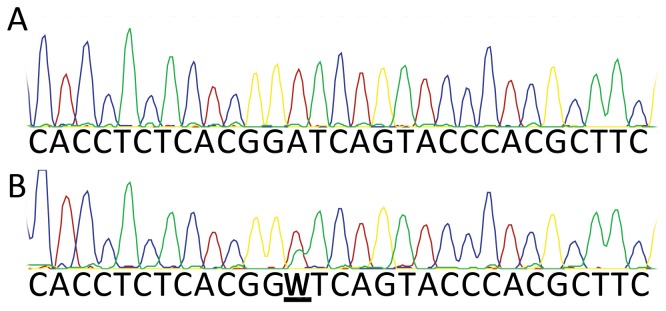
Synthetic MEST template mixing experiments using marked templates. PCR with primers MESTPF1/MESTPR3C or MESTPF1A/MESTPR3C on the two gBlock constructs (wild-type and mutant) generated synthetic templates that were identical except for the presence of one variant base introduced with the mismatch primer MESTPF1A. Methylated and unmethylated forms of these amplicons were diluted and mixed, subjected to PCR, and then genotyped by Sanger sequencing. Products derived from the synthetic templates could be distinguished due to the presence of either an A or T at this position. (A) wild-type templates for which the “T” allele was methylated, and the “A” allele was unmethylated, showing apparent “A” homozygosity; (B) mutant (non-G4 forming) templates for which the “T” allele was methylated, and the “A” allele was unmethylated, showing apparent heterozygosity (W).

To further investigate the role of methylation in allelic drop-out, the two synthetic templates (wild-type and the mutant lacking G4-forming ability) were amplified with two pairs of primers, MESTPF1/MESTPR3C and MESTPF1A/MESTPR3C (Table S1). MESTPF1A incorporated a novel variant base that generated an artificial haplotype distinguishable by the presence of this variant nucleotide. Template mixing experiments followed by PCR and Sanger sequencing, similar to those described above, were then repeated (Figure 7). Mixing of methylated and unmethylated wild-type templates resembled the situation for genomic DNA and resulted in consistent drop-out of the methylated strand. Mixing of methylated and unmethylated mutant templates, neither of which can form G4s, resulted in occasional allelic drop-out of the methylated strand, however a peak indicative of the other haplotype was always visible (Figure 7).

### Tm comparison of methylated versus unmethylated templates

To explore potential *T_m_* differences between methylated and unmethylated templates, we used HRM analysis on an Eco real time PCR instrument (Illumina, San Diego, CA) with Syto 9 dye (Invitrogen, Auckland, New Zealand). Primers PF2/PR3C were used to amplify a 358 bp region which had a GC content of 66.5% and contained 24 CG dinucleotides. *In vitro* methylation was carried out as described above. In deionized water we consistently found *T_m_* of the methylated DNA to be 91.6°C and the unmethylated DNA to be 90.6°C. When this was repeated in PCR buffer (Roche, Mannheim, Germany) the *T_m_* increased to 92.3°C for methylated and 91.4°C for unmethylated product.

## Discussion

This paper describes a region of the human genome that displays unusual properties *in vitro*. The ∼500 bp region of DNA located in the CpG island and promoter region of the maternally imprinted gene *MEST* shows a surprising form of allelic drop-out during PCR, whereby one allele from every subject analysed consistently fails to amplify. Three SNPs in this region were in complete linkage disequilibrium, defining two human haplotypes, and these polymorphisms allowed us to observe the pattern of allelic loss in this region. Different alleles (as defined by the three SNPs) were prone to drop-out in different individuals (Table S2).

### Two potential contributors to allelic drop-out

We hypothesised that two features of the *MEST* promoter region were contributing to allelic drop-out. The first was that *MEST* is an imprinted gene and one allele is heavily methylated at CpG dinucleotides [31], [32]. The second feature was the presence of several G-rich elements that were predicted to form G4 structures, which can interfere with DNA polymerase progression and DNA replication [13], [14]. We tested our hypothesis by exploring the nature of the *MEST* G4 structures, determining which allele was lost from PCR, and comparing the individual and additive effects of G4 formation versus cytosine methylation on allelic drop-out.

### Cluster of G4s in imprinted MEST promoter

Examination of the *MEST* promoter region using various computer algorithms revealed the presence of at least three G-rich motifs on one strand, which fitted the canonical G4 motif. CD spectroscopy on oligonucleotides corresponding to these regions (G4MEST1-3) showed that all were capable of forming structures in the presence of K^+^ with CD signatures characteristic of G4s (Figure 2). G4MEST 1 and 2 gave signatures that suggested structurally mixed species containing both parallel and anti-parallel G-quadruplex forms. CD spectroscopy further demonstrated that in standard buffer/salt mixes used for PCR, containing Tris, MgCl_2_ and KCl, both G4MEST1 and 3 shifted to a predominantly parallel form, and that these conditions resulted in significant stabilisation of all three structures, with G4MEST2 and G4MEST3 showing a *T*_m_ of >99.0°C in PCR buffer (Table 1 and Figure 3). The structural potential of G4MEST1-3 was confirmed by native PAGE, which showed that the G4-forming oligonucleotides migrated more rapidly than equivalent oligonucleotides with T residues substituted for critical G residues likely to be involved in G4 formation (Figure 5). Staining with SYBR Safe (Life Technologies, Carlsbad,CA, USA) proved effective at revealing oligonucleotides capable of forming G4 structures, including potentially higher order aggregate structures that formed for G4MEST2 and G4MEST3.

### Consistent drop-out of maternal allele in PCR

In order to understand the nature of the allelic drop-out phenomenon at this locus, we applied four approaches to determine which allele was prone to drop-out. Our working hypothesis suggested that the maternal, methylated allele would be consistently lost during PCR. Because it was difficult to study this phenomenon in genomic DNA where one allele was utterly resistant to amplification, we first modelled the situation using PCR products representing one or other *MEST* haplotype (ATA or GCG) generated from different subjects. These products lack methylation but can be distinguished by their genotype. Each allele was subjected to *in vitro* methylation using M.SssI, to generate four templates representing those expected in nature, i.e. methylated or unmethylated ATA, and methylated or unmethylated GCG. Experiments where different pairs of templates were mixed in equal quantity clearly showed that the methylated template would always “lose out” to the unmethylated template in PCR (Figure S4).

Second, we developed a modified PCR buffer that contained low concentrations of sodium phosphate and magnesium chloride, and lacked potassium altogether. We found this was effective only for amplification of short regions of DNA, so we targeted it at a 218bp region containing two of the three *MEST* promoter SNPs, 15 CpG dinucleotides, and only one of the G4 regions, G4MEST1, which had the lowest *T_m_* of the three G4s (Figure 1). Using this method, we were able to effectively genotype genomic DNA samples, and we identified three informative parent-offspring trios that enabled parent-of-origin analysis of the allelic drop-out. By comparing the genotypes obtained with this modified PCR versus a standard PCR for these three trios, it was clear that the maternal, methylated allele failed to amplify in standard PCR (Figure S3).

Third, in order to extend this initial result we developed a methylation-specific PCR that could distinguish the maternal (methylated) and paternal (unmethylated) *MEST* alleles. By applying this method to bisulfite treated DNA from many samples, including the trios described above, we confirmed that the methylated allele consistently dropped out of the assay.

The fourth and final proof that the maternal, methylated allele was being lost depended on treatment of genomic DNA prior to PCR with either McrBC endonuclease, which specifically cuts DNA only when it contains methylated cytosines on one or both strands, or the methylation-sensitive restriction enzyme HpaII. By pre-treating genomic DNA of known haplotypes, followed by standard PCR amplification we were able to individually genotype each allele. This further confirmed that the methylated allele was failing to amplify under standard PCR protocol.

Taken together, these results prove that the methylated, maternal allele of the *MEST* promoter region is refractory to amplification under standard PCR conditions and suffers allelic drop-out. This allelic drop-out can only be circumvented by extraordinary measures, such as using a modified, potassium free reaction buffer and targeting a very short amplicon in this region.

### Effect of CpG methylation on G4 properties

Having established which allele was failing to amplify in PCR, we set out to explore the relative contribution of cytosine methylation and G4 formation to this phenomenon. G4 structures formed by oligonucleotides containing methylated CpG residues display increased stability compared to equivalent unmethylated oligonucleotides [20]. Therefore we evaluated the influence of CpG methylation on formation of the three *MEST* promoter G4. A set of oligonucleotides corresponding to G4MEST1-3 was synthesised, each containing a single methylated CpG dinucleotide (named G4MEST1LM, 2 M and 3 M) and these were studied by CD spectroscopy (Figure 4). G4MEST1LM was the sole methylated oligonucleotide to adopt G4 conformation in both NaPi and PCR buffer, with the trough at 245 nm and peak at 260 nm with a shoulder to ∼290 nm indicating the presence of mixed G4 conformations. These observations may be explained by the potential of this relatively long region to form more than one G4 structure. The structural signatures of G4MEST2M and G4MEST3M were comparable to those observed for their unmethylated counterparts in PCR but not in NaPi buffer. Further investigation showed that similar signatures could also be generated in phosphate buffer with the addition of MgCl_2_, however *T*_m_ was greatly increased in PCR buffer (Table 1). On PAGE performed in TBE buffer the methylated G4MEST2M and G4MEST3M oligonucleotides migrated with a similar mobility to their unmethylated counterparts, and they strongly stained with the intercalating dye SYBR Safe (Figure 5).

### G4 formation and CpG methylation both contribute to maternal allele drop-out

We then set out to empirically evaluate the respective impact on allelic drop-out of either cytosine methylation or G4 formation. We designed two synthetic templates spanning the three *MEST* promoter SNPs one of which contained multiple substitutions to eradicate the three G4 forming motifs. *In vitro* methylation and template mixing experiments followed by PCR and genotyping with Sanger sequencing were then systematically carried out. This demonstrated that templates containing the three *MEST* G4 were more difficult to amplify during PCR than equivalent mutant (non-G4) templates regardless of methylation status (Figure S4). Furthermore, when methylated and unmethylated mutant templates were mixed, both alleles could be detected (Figure 7). These observations strongly suggest that both secondary structure formation and CpG methylation are important for the observed allelic drop-out, and that neither property alone is sufficient to fully account for the observed results. This conclusion is reinforced by the observation that the allelic dropout is highly dependent on the presence of K^+^ ions.

When 7 deaza dGTP was used as a substitute for dGTP under standard PCR conditions no influence on genotyping results were observed. However, when 7 deaza dGTP was incorporated into the template DNA and genotyping was carried out using standard PCR, this alleviated the methylation specific allelic drop-out. This indicates the drop-out occurs as a result of Hoogsteen base pairing present in the DNA template, as would be required for G4, and is not a property of nascent strands generated during PCR.

CpG methylation is known to increase the *T_m_* of DNA [39]–[41], and indeed this was attributed as the reason for a less persistent allelic drop-out in another region of *MEST*, adjacent to the region reported in this paper [37](Figure 1). Our *in vitro* HRM observations suggest that the *T_m_* difference between methylated and unmethylated templates in the *MEST* promoter region is minimal, and unlikely to be a major contributor to allelic drop-out. This raises the question of whether there is a more complex interaction between cytosine methylation and G4 structure. Hardin (1991) showed that G4 containing methylated CpG had higher stability *in vitro* [20], and another more recent *in vitro* analysis suggested that CpG methylation can greatly increase the thermal stability of a G4 that forms in the P1 promoter of the oncogene *bcl-2* [23]. If anything, however, methylated *MEST* G4 oligonucleotides were less thermally stable than their unmethylated equivalents and their formation was dependent on the presence of MgCl_2_ (Figure 4 and Table 1), suggesting that increased thermal stability due to methylation is unlikely to explain the *MEST* allelic drop-out.

### Extending the range of known mechanisms for allelic drop-out in PCR

This work has revealed a novel mechanism for allelic drop-out that leads to a parent-of-origin specific bias in PCR. The allelic drop-out results from the combined impact of G4 formation and presence of differential allelic methylation at the *MEST* locus. The role of potassium-dependent structures in disrupting PCR and DNA sequencing has long been recognised [42], and G4 structures have been implicated in the preferential allelic amplification of a G-rich microsatellite allele [13]. More recent reports have attributed PCR allelic drop-out in the clinical diagnostic setting to the variable effects of single nucleotide variants on formation of G4 structures [15], [16], or to the presence of cytosine methylation in an imprinted gene [38]. Similarly, Bunyan et al. (2011) [37] described differential allelic amplification of a region of *MEST* that is adjacent to but not overlapping the cluster of G4 we observed (Figure 1), which they attributed to the higher *T_m_* of the methylated allele. This drop-out was overcome by the relatively trivial step of using a longer denaturation stage prior to addition of the polymerase [37]. There appear to be no prior reports that document parent-of-origin specific allelic drop-out, or that attribute drop-out to the combined effects of G4 formation and cytosine methylation.

## Conclusions

In summary, we describe a small region located in the CpG island and promoter of the maternally imprinted gene *MEST*, which displays marked allelic drop-out during PCR. Bi-allelic amplification at this locus could only be achieved with extraordinary effort and experimental manipulation. We show that it is always the maternal, methylated allele which fails to amplify, and that this phenomenon is associated with G4 formation and cytosine methylation in this region. It is possible that this type of parent-of-origin specific allelic loss could occur at other loci, and represents a potential concern for molecular diagnostic analyses and research studies involving imprinted genes.

## Supporting Information

Figure S1 **Synthetic plasmid constructs for** ***MEST*** **promoter region.** Synthetic plasmid constructs (IDT Pte. Ltd., Singapore) were generated containing 636 bp of sequence corresponding to chr7:130,131,385-130,132,020 (hg19), and encompassing all three SNPs (indicated in bold) and all three quadruplex forming regions (G4MEST1-3 are highlighted with grey shading, and the extended G4MEST1L region is shown with darker grey shading). One of these constructs represented genomic sequence (of ATA haplotype) as illustrated, and the other was modified with 38 G to T substitutions (underlined bases) to remove all potential G4 forming ability.
(DOCX)

Figure S2 **DNA mixing experiment showing “mock heterozygosity”.** PCR of the MEST promoter region was carried out using genomic DNA from two GODS subjects and amplicons were genotyped by Sanger sequencing. (A) GODS subject #2 showing apparent ATA homozygosity; (B) GODS subject #13 showing apparent GCG homozygosity; (C) equal concentration of GODS subject #2 and #13 DNA mixed, showing apparent GCG/ATA heterozygosity.
(DOCX)

Figure S3 **Informative family trios.** GODS probands were screened by traditional PCR to identify those with an apparent ATA genotype. Because ATA is the minor allele in the population, our assumption was that most of these cases would prove to be heterozygotes, with the major GCG haplotype obscured due to allelic drop-out. Using the short PCR amplicon (218 bp) in potassium-free PCR buffer we were able to accurately genotype all members of the trios. These data showed that all offspring were indeed heterozygous ATA/GCG, as were all fathers, and that the mothers in these trios were all GCG homozygotes. The figure illustrates for each GODS subject (identified by four digit study numbers) the true *MEST* promoter haplotype, and for each of the three probands the observed genotype generated by traditional PCR. For all three trios it is clear that one maternal GCG allele has dropped out of the traditional PCR.
(DOCX)

Figure S4 **Synthetic** ***MEST*** **template mixing experiments using mutated vs. wild-type templates.** The G4 forming region (G4MEST1L) is indicated as a grey bar above sequence traces. Wild-type *MEST* sequence is illustrated at bottom of figure. SNP rs75098511 is underlined. (A). Methylated wild-type vs. unmethylated mutant; (B). Methylated mutant vs. unmethylated wild-type; (C). Methylated wild-type vs. methylated mutant; (D). Unmethylated wild-type vs. unmethylated mutant.
(DOCX)

Table S1 **Oligonucleotide sequences used in this study.**
(DOCX)

Table S2 **Genotyping results for GODS subjects.**
(DOCX)
