# Correction: G-Quadruplex Structures and CpG Methylation Cause Drop-Out of the Maternal Allele in Polymerase Chain Reaction Amplification of the Imprinted *MEST* Gene Promoter

**DOI:** 10.1371/journal.pone.0122940

**Published:** 2015-03-26

**Authors:** The PLOS ONE Staff

In the Author Contributions section, Martin Kennedy (MAK) should be listed as one of the persons who conceived and designed the experiments.

## References

[1] Stevens AJ, Stuffrein-Roberts S, Cree SL, Gibb A, Miller AL, Doudney K, et al (2014) G-Quadruplex Structures and CpG Methylation Cause Drop-Out of the Maternal Allele in Polymerase Chain Reaction Amplification of the Imprinted *MEST* Gene Promoter. PLoS ONE 9(12): e113955 doi: 10.1371/journal.pone.0113955 25437198 10.1371/journal.pone.0113955 PMC4249981

