# Supplementary material for: G-Quadruplex Structures and CpG Methylation Cause Drop-Out of the Maternal Allele in Polymerase Chain Reaction Amplification of the Imprinted MEST Gene Promoter
Source: PLoS One. 2014 Dec 1;9(12):e113955. doi: 10.1371/journal.pone.0113955 (PMC4249981; doi:10.1371/journal.pone.0113955)
Supplement: Figure S1 — Synthetic plasmid constructs for MEST promoter region. Synthetic plasmid constructs (IDT Pte. Ltd., Singapore) were generated containing 636 bp of sequence corresponding to chr7:130,131,385-130,132,020 (hg19), and encompassing all three SNPs (indicated in bold) and all three quadruplex forming regions (G4MEST1-3 are highlighted with grey shading, and the extended G4MEST1L region is shown with darker grey shading). One of these constructs represented genomic sequence (of ATA haplotype) as illustrated, and the other was modified with 38 G to T substitutions (underlined bases) to remove all potential G4 forming ability. (DOCX) [file pone.0113955.s001.docx]

**Figure S1. Synthetic plasmid constructs for *MEST* promoter region**. Synthetic plasmid constructs (IDT Pte. Ltd., Singapore) were generated containing 636bp of sequence corresponding to chr7:130,131,385-130,132,020 (hg19), and encompassing all three SNPs (indicated in bold) and all three quadruplex forming regions (G4MEST1-3 are highlighted with grey shading, and the extended G4MEST1L region is shown with darker grey shading). One of these constructs represented genomic sequence (of ATA haplotype) as illustrated, and the other was modified with 38 G to T substitutions (underlined bases) to remove all potential G4 forming ability.
