# Supplementary material for: G-Quadruplex Structures and CpG Methylation Cause Drop-Out of the Maternal Allele in Polymerase Chain Reaction Amplification of the Imprinted MEST Gene Promoter
Source: PLoS One. 2014 Dec 1;9(12):e113955. doi: 10.1371/journal.pone.0113955 (PMC4249981; doi:10.1371/journal.pone.0113955)
Supplement: Figure S2 — DNA mixing experiment showing “mock heterozygosity”. PCR of the MEST promoter region was carried out using genomic DNA from two GODS subjects and amplicons were genotyped by Sanger sequencing. (A) GODS subject #2 showing apparent ATA homozygosity; (B) GODS subject #13 showing apparent GCG homozygosity; (C) equal concentration of GODS subject #2 and #13 DNA mixed, showing apparent GCG/ATA heterozygosity. (DOCX) [file pone.0113955.s002.docx]

**Figure S2.** **DNA mixing experiment showing “mock heterozygosity”.**  PCR of the MEST promoter region was carried out using genomic DNA from two GODS subjects and amplicons were genotyped by Sanger sequencing. (A) GODS subject #2 showing apparent ATA homozygosity; (B) GODS subject #13 showing apparent GCG homozygosity; (C) equal concentration of GODS subject #2 and #13 DNA mixed, showing apparent GCG/ATA heterozygosity.
