# Supplementary material for: G-Quadruplex Structures and CpG Methylation Cause Drop-Out of the Maternal Allele in Polymerase Chain Reaction Amplification of the Imprinted MEST Gene Promoter
Source: PLoS One. 2014 Dec 1;9(12):e113955. doi: 10.1371/journal.pone.0113955 (PMC4249981; doi:10.1371/journal.pone.0113955)

**Figure S3.** **Informative family trios**. GODS probands were screened by traditional PCR to identify those with an apparent ATA genotype. Because ATA is the minor allele in the population, our assumption was that most of these cases would prove to be heterozygotes, with the major GCG haplotype obscured due to allelic drop-out. Using the short PCR amplicon (218bp) in potassium-free PCR buffer we were able to accurately genotype all members of the trios. These data showed that all offspring were indeed heterozygous ATA/GCG, as were all fathers, and that the mothers in these trios were all GCG homozygotes. The figure illustrates for each GODS subject (identified by four digit study numbers) the true *MEST* promoter haplotype, and for each of the three probands the observed genotype generated by traditional PCR. For all three trios it is clear that one maternal GCG allele has dropped out of the traditional PCR.


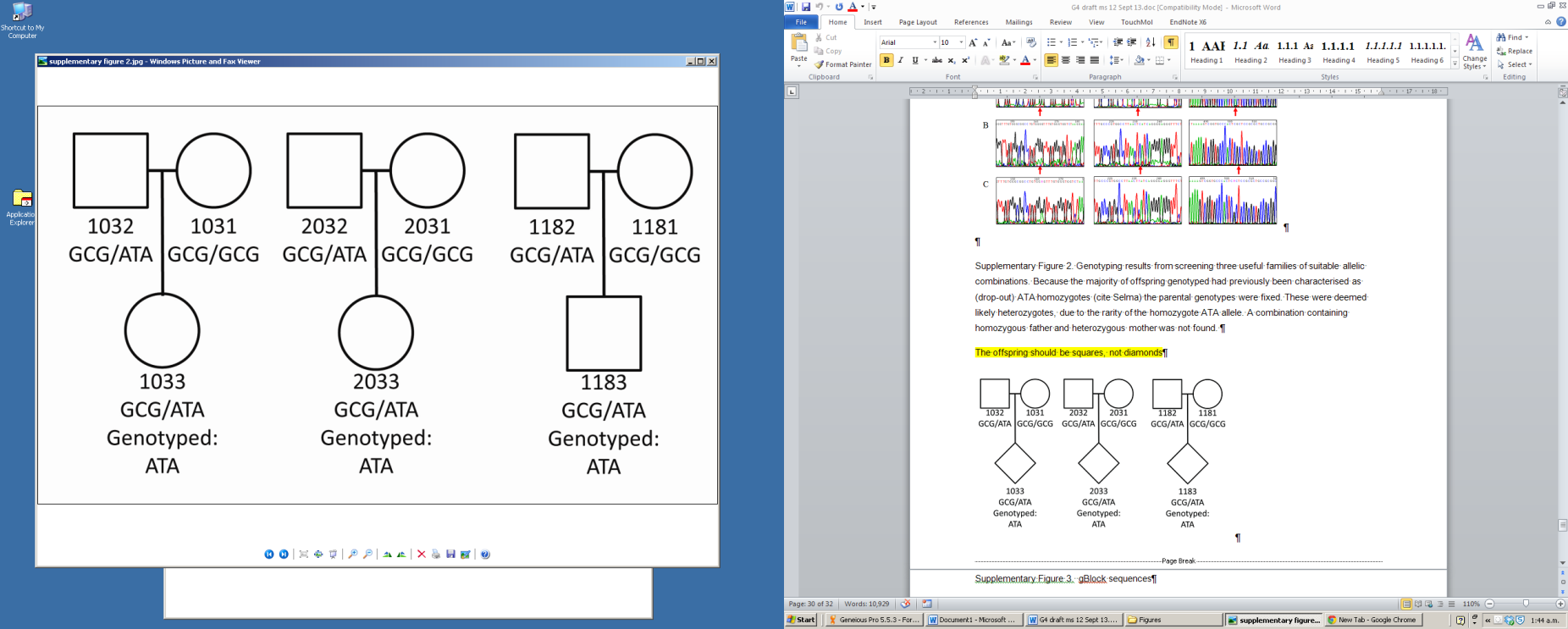

Supplement: Figure S3 — Informative family trios. GODS probands were screened by traditional PCR to identify those with an apparent ATA genotype. Because ATA is the minor allele in the population, our assumption was that most of these cases would prove to be heterozygotes, with the major GCG haplotype obscured due to allelic drop-out. Using the short PCR amplicon (218 bp) in potassium-free PCR buffer we were able to accurately genotype all members of the trios. These data showed that all offspring were indeed heterozygous ATA/GCG, as were all fathers, and that the mothers in these trios were all GCG homozygotes. The figure illustrates for each GODS subject (identified by four digit study numbers) the true MEST promoter haplotype, and for each of the three probands the observed genotype generated by traditional PCR. For all three trios it is clear that one maternal GCG allele has dropped out of the traditional PCR. (DOCX) [file pone.0113955.s003.docx]
