# Supplementary material for: G-Quadruplex Structures and CpG Methylation Cause Drop-Out of the Maternal Allele in Polymerase Chain Reaction Amplification of the Imprinted MEST Gene Promoter
Source: PLoS One. 2014 Dec 1;9(12):e113955. doi: 10.1371/journal.pone.0113955 (PMC4249981; doi:10.1371/journal.pone.0113955)

**Figure S4.** **Synthetic *MEST* template mixing experiments using mutated vs. wild-type templates.** The G4 forming region (G4MEST1L) is indicated as a grey bar above sequence traces. Wild-type *MEST* sequence is illustrated at bottom of figure. SNP rs75098511 is underlined. (A). Methylated wild-type vs. unmethylated mutant; (B). Methylated mutant vs. unmethylated wild-type; (C). Methylated wild-type vs. methylated mutant; (D). Unmethylated wild-type vs. unmethylated mutant.


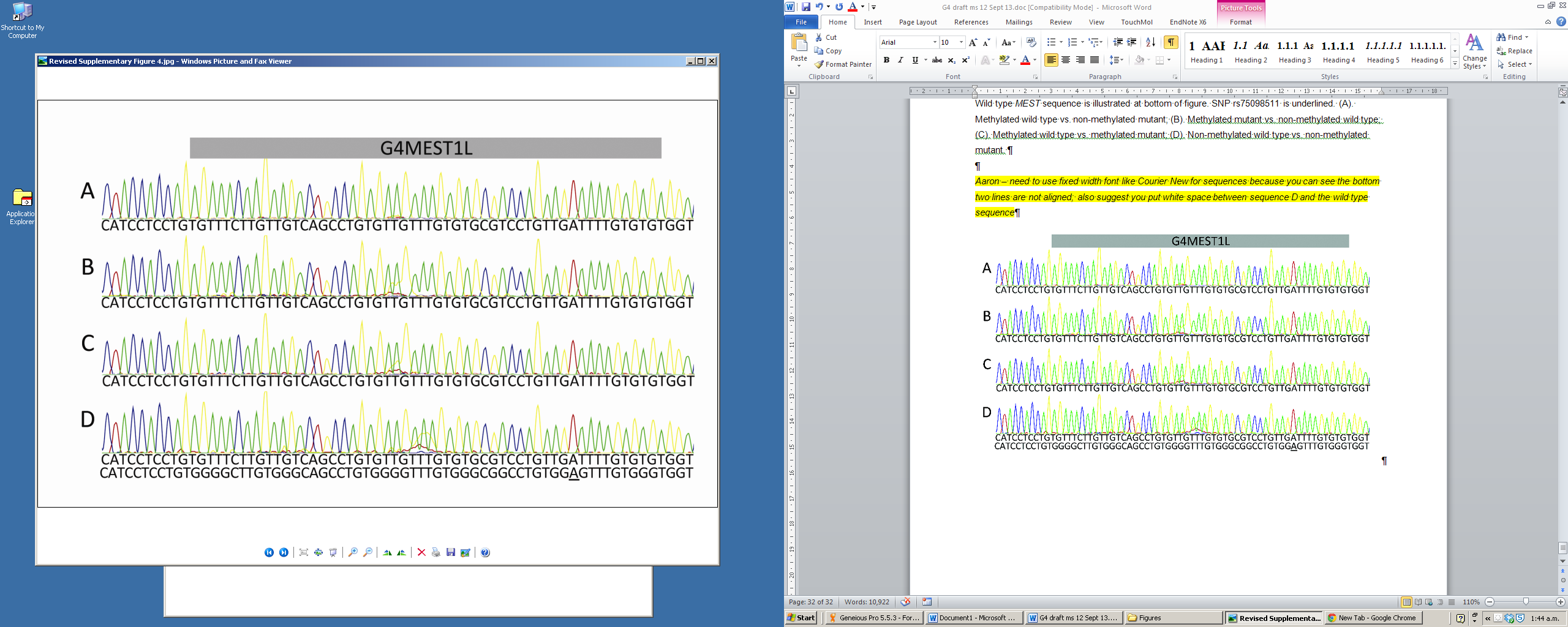

Supplement: Figure S4 — Synthetic MEST template mixing experiments using mutated vs. wild-type templates. The G4 forming region (G4MEST1L) is indicated as a grey bar above sequence traces. Wild-type MEST sequence is illustrated at bottom of figure. SNP rs75098511 is underlined. (A). Methylated wild-type vs. unmethylated mutant; (B). Methylated mutant vs. unmethylated wild-type; (C). Methylated wild-type vs. methylated mutant; (D). Unmethylated wild-type vs. unmethylated mutant. (DOCX) [file pone.0113955.s004.docx]
