# Supplementary material for: G-Quadruplex Structures and CpG Methylation Cause Drop-Out of the Maternal Allele in Polymerase Chain Reaction Amplification of the Imprinted MEST Gene Promoter
Source: PLoS One. 2014 Dec 1;9(12):e113955. doi: 10.1371/journal.pone.0113955 (PMC4249981; doi:10.1371/journal.pone.0113955)
Supplement: Table S1 — Oligonucleotide sequences used in this study. (DOCX) [file pone.0113955.s005.docx]

**Table S1. Oligonucleotide sequences used in this study.**

| **Name** | **Sequence** | **Purpose** |
| --- | --- | --- |
| MESTPF1 | CGTGGCTCGCCACCTCTCAC | PCR primer |
| MESTPF1A* | CGTGGCTCGCCACCTCTCACGGTTCAGTACCCAC | Mismatch primer |
| MESTPR2 | CGTTAACCGCCAACCCTGAG | PCR primer |
| MESTPF3 | CCCAGAGCCCTGCTGCCCCTTAG | PCR primer |
| MESTPR3 | AGTGGGCACCGACTTTTAGAG | PCR primer |
| MESTPR3C | TGCCGCAGAGGAGGTGCC | PCR primer |
| MESTPR4 | CGTTAACCGCCAACCCTGAG | PCR primer |
| MESTPR6 | CGCCCAGAGGCAGCCCCAGCT | PCR primer |
| MESTPF5 | GCGGCGAGCAAGGGAGCAGG | PCR primer |
| G4MEST1 | GGGCTTGTGGGCAGCCTGTGGGGTTTGTGG | For G4 physical analyses |
| G4MEST1L | GGGCTTGTGGGCAGCCTGTGGGGTTTGTGGGCGGCCTGTGGAGTTTGTGGG | For G4 physical analyses |
| G4MEST2 | GGGAGCAGCGGGGTCTTGGGGAGGGGG | For G4 physical analyses |
| G4MEST3 | GGGCGGGCTAGGGGCGGGGCGCGGGTGGG | For G4 physical analyses |
| G4MEST1A | GAACTTGTGAACAGCCTGTGGAATTTGTGA | Mutated G4 control |
| G4MEST2A | GAGAGCAGCGAAGTCTTGAAGAGAAAG | Mutated G4 control |
| G4MEST3A | GAGCGAGCTAGAAGCGAAGCGCGAGTGAG | Mutated G4 control |
| G4MEST1LM** | GGGCTTGTGGGCAGCCTGTGGGGTTTGTGGGCGGCCTGTGGAGTTTGTGGG | Methylated oligonucleotide |
| G4MEST2M** | GGGAGCAGCGGGGTCTTGGGGAGGGGG | Methylated oligonucleotide |
| G4MEST3M** | GGGCGGGCTAGGGGCGGGGCGCGGGTGGG | Methylated oligonucleotide |
| BSPF1CG | AAGGAAATTTGTTTCGCG | Methylated allele bisulphite PCR |
| BSPF1TG | TGTAAAGGAAATTTGTTTTGTG | Non-methylated allele bisulphite PCR |
| BSPMESTR3 | CAAAAAAAATACC(A/G)AAATATACT | Bisulphite reverse primer |

* Underlining indicates penultimate base mismatch.

**Underlining indicates methylated cytosine.
