# Supplementary material for: G-Quadruplex Structures and CpG Methylation Cause Drop-Out of the Maternal Allele in Polymerase Chain Reaction Amplification of the Imprinted MEST Gene Promoter
Source: PLoS One. 2014 Dec 1;9(12):e113955. doi: 10.1371/journal.pone.0113955 (PMC4249981; doi:10.1371/journal.pone.0113955)
Supplement: Table S2 — Genotyping results for GODS subjects. (DOCX) [file pone.0113955.s006.docx]

**Table S2. Genotyping results for GODS subjects.**

| **Sample** | **rs75098511*** | **rs73724326*** | **rs116603785*** | **rs1050582**** | **rs10863**** |
| --- | --- | --- | --- | --- | --- |
| **1** | GG | CC | GG | CG | GG |
| **2** | AA | TT | AA | CG | GG |
| **3** | GG | CC | GG | CC | GG |
| **4** | GG | CC | GG | CC | GG |
| **5** | GG | CC | GG | CG | GG |
| **6** | GG | CC | GG | CG | GA |
| **7** | GG | CC | GG | CG | GA |
| **8** | GG | CC | GG | GG | GA |
| **9** | GG | CC | GG | CG | GG |
| **10** | GG | CC | GG | CG | GA |
| **11** | GG | CC | GG | CG | AA |
| **12** | GG | CC | GG | CC | GG |
| **13** | GG | CC | GG | CC | GG |
| **14** | GG | CC | GG | CG | GA |
| **15** | GG | CC | GG | CC | GG |
| **16** | GG | CC | GG | CG | GG |
| **17** | GG | CC | GG | CC | GG |
| **18** | GG | CC | GG | CC | GG |
| **19** | GG | CC | GG | CC | GG |
| **20** | AA | TT | AA | CG | GG |
| **21** | GG | CC | GG | CG | GG |
| **22** | GG | CC | GG | CC | GG |
| **23** | AA | TT | AA | CG | GG |

* SNPs in the promoter region

** SNPs in the 3’untranslated region
